# Targeted single-nucleus sequencing of 39,800 neurons reveals extensive low-frequency somatic variants

**DOI:** 10.64898/2026.09.21.753260

**Published:** 2026-09-22

**Authors:** Vanshika Bidhan, Sarah Wynants, Toon Swings, Cristina T. Vicente, Fahri Küçükali, Marleen Van den Broeck, Jeroen GJ. van Rooij, Merel O. Mol, Laura L. Donker Kaat, Safa Al-Sarraj, Istvan Bodi, Andrew King, Claire Troakes, Christos Proukakis, Jolien Schaeverbeke, Dietmar R. Thal, Rik Vandenberghe, Mathieu Vandenbulcke, Aivi T. Nguyen, R. Ross Reichard, Julia Kofler, Oscar L. Lopez, Charles L. White, Bradley F. Boeve, Neill R. Graff-Radford, Keith A. Josephs, Ronald C. Petersen, Nikhil B. Ghayal, Melissa E. Murray, Dennis W. Dickson, John C. van Swieten, Kristel Sleegers, Harro Seelaar, Wouter De Coster, Rosa Rademakers

## Abstract

Recent evidence implicates somatic mutations in disease-associated genes across multiple neurodegenerative disorders, including the identification of two somatic *TARDBP* variants as a putative cause of frontotemporal lobar degeneration with TDP-43 pathology type C (FTLD-TDP type C). However, these findings remain anecdotal.

We performed single-nucleus amplicon sequencing of 39,800 neurons from the superior temporal gyrus of sporadic FTLD-TDP type C patients (34,738 neurons, 52 individuals) and non-demented controls (5,062 neurons, 26 individuals) using the Mission Bio Tapestri platform and investigated somatic variants in *TARDBP* and other FTLD-TDP-associated genes in a case-control setting.

We uncovered an extensive landscape of ultra-low-frequency (<1%) somatic mutations across all analysed genes, including *TARDBP* . Rare somatic occurrences of known ALS/FTD-associated germline *TARDBP* variants, including p.N267S and p.M337V, were identified exclusively in patients. Among all targeted genes, *TARDBP* had the highest proportion of neurons harbouring a somatic variant, and this mutational burden was lower in individuals who died at an older age. Strikingly, the C-terminal domain of TDP-43, where most ALS/FTD germline pathogenic variants reside, exhibited a lower neuronal mutational burden than its other domains. This suggests that neurons harbouring damaging mutations may undergo progressive loss prior to autopsy, leaving only rare survivors at frequencies too low to detect by bulk or small-scale single-cell approaches. Together, our findings provide an unprecedented resolution into the somatic mutational landscape of post-mitotic neurons in FTLD-TDP type C and reveal a gene-specific layer of neuronal mosaicism that current single-cell WGS studies fail to detect.

## Full Text Availability

The license terms selected by the author(s) for this preprint version do not permit archiving in PMC. The full text is available from the preprint server.

